# Cortical and subcortical morphological alterations in motor subtypes of Parkinson’s disease

**DOI:** 10.1038/s41531-022-00435-3

**Published:** 2022-12-05

**Authors:** Jianyu Li, Yuanchao Zhang, Zitong Huang, Yihan Jiang, Zhanbing Ren, Daihong Liu, Jiuquan Zhang, Roberta La Piana, Yifan Chen

**Affiliations:** 1 Key Laboratory for NeuroInformation of Ministry of Education, School of Life Science and Technology, University of Electronic Science and Technology of China, Chengdu, 610054 P. R. China; 2 Department of Physical Education, Shenzhen University, Shenzhen, 518060 China; 3 Department of Radiology, Chongqing University Cancer Hospital & Chongqing Cancer Institute & Chongqing Cancer Hospital, Chongqing, 400030 P. R. China; 4 Department of Neurology & Neurosurgery, Montreal Neurological Institute and Hospital, McGill University, Montreal, QC H3A 0G4 Canada

**Keywords:** Parkinson's disease, Magnetic resonance imaging

## Abstract

Parkinson’s disease (PD) can be classified into an akinetic-rigid (AR) and a tremor-dominant (TD) subtype based on predominant motor symptoms. Patients with different motor subtypes often show divergent clinical manifestations; however, the underlying neural mechanisms remain unclear. This study aimed to characterize the cortical and subcortical morphological alterations in motor subtypes of PD. T1-weighted MRI images were obtained for 90 patients with PD (64 with the AR subtype and 26 with the TD subtype) and 56 healthy controls (HCs). Cortical surface area, sulcal depth (measured by Freesurfer’s Sulc index), and subcortical volume were computed to identify the cortical and subcortical morphological alterations in the two motor subtypes. Compared with HCs, we found widespread surface area reductions in the AR subtype yet sparse surface area reductions in the TD subtype. We found no significant Sulc change in the AR subtype yet increased Sulc in the right supramarginal gyrus in the TD subtype. The hippocampal volumes in both subtypes were lower than those of HCs. In PD patients, the surface area of left posterior cingulate cortex was positively correlated with Mini-Mental State Examination (MMSE) score, while the Sulc value of right middle frontal gyrus was positively correlated with severity of motor impairments. Additionally, the hippocampal volumes were positively correlated with MMSE and Montreal Cognitive Assessment scores and negatively correlated with severity of motor impairments and Hoehn & Yahr scores. Taken together, these findings may contribute to a better understanding of the neural substrates underlying the distinct symptom profiles in the two PD subtypes.

## Introduction

Parkinson’s disease (PD) is a highly heterogeneous neurodegenerative disorder characterized by a broad spectrum of motor symptoms such as tremor, akinesia/bradykinesia, and rigidity, as well as diverse non-motor symptoms^1^. Based on the predominant motor symptoms, PD patients can be classified into two major subtypes i.e., the tremor-dominant (TD) and the akinetic-rigid (AR) subtypes^2^. In addition to the motor symptoms, the two subtypes also differ in their non-motor symptoms and outcomes. For example, the TD subtype is associated with a lower degree of cognitive impairments and minor incidence of neuropsychiatric symptoms, whereas the AR subtype with poorer prognosis and more common dementia^3,4^, pointing to distinct neural substrates in the two motor subtypes.

A number of neuroimaging studies have reported divergent functional brain alterations in the AR and TD subtypes^5–7^, whereas few studies have examined the structural brain alterations in the two subtypes. In particular, using voxel-based morphometry, a structural MRI study showed decreased cerebellar gray matter in the TD subtype but not AR^8^. Using surface-based morphometry, our group showed similar cortical thickness reductions yet distinct cortical gyrification patterns in the two subtypes^9^. However, the patterns of cortical surface area and sulcal depth, two important features that carry unique morphological information of the cerebral cortex, remain unexplored in the two subtypes. Investigation of these two morphological features may shed some new light on neural substrates underlying their distinct clinical manifestations.

Using surface-based local surface area and Sulc (a measure of sulcal depth) maps^10^, the present study aims to characterize the cortical surface area and sulcal depth patterns in patients with the AR and TD subtypes compared with HCs. Meanwhile, we also examined the subtype-specific alterations in the volumes of subcortical structures. Based on existing literature, we hypothesized that the AR subtype would show more widespread surface area and sulcal depth changes compared with the TD subtype.

## Results

### Surface area changes in AR and TD subtypes

**Fig. 1:**
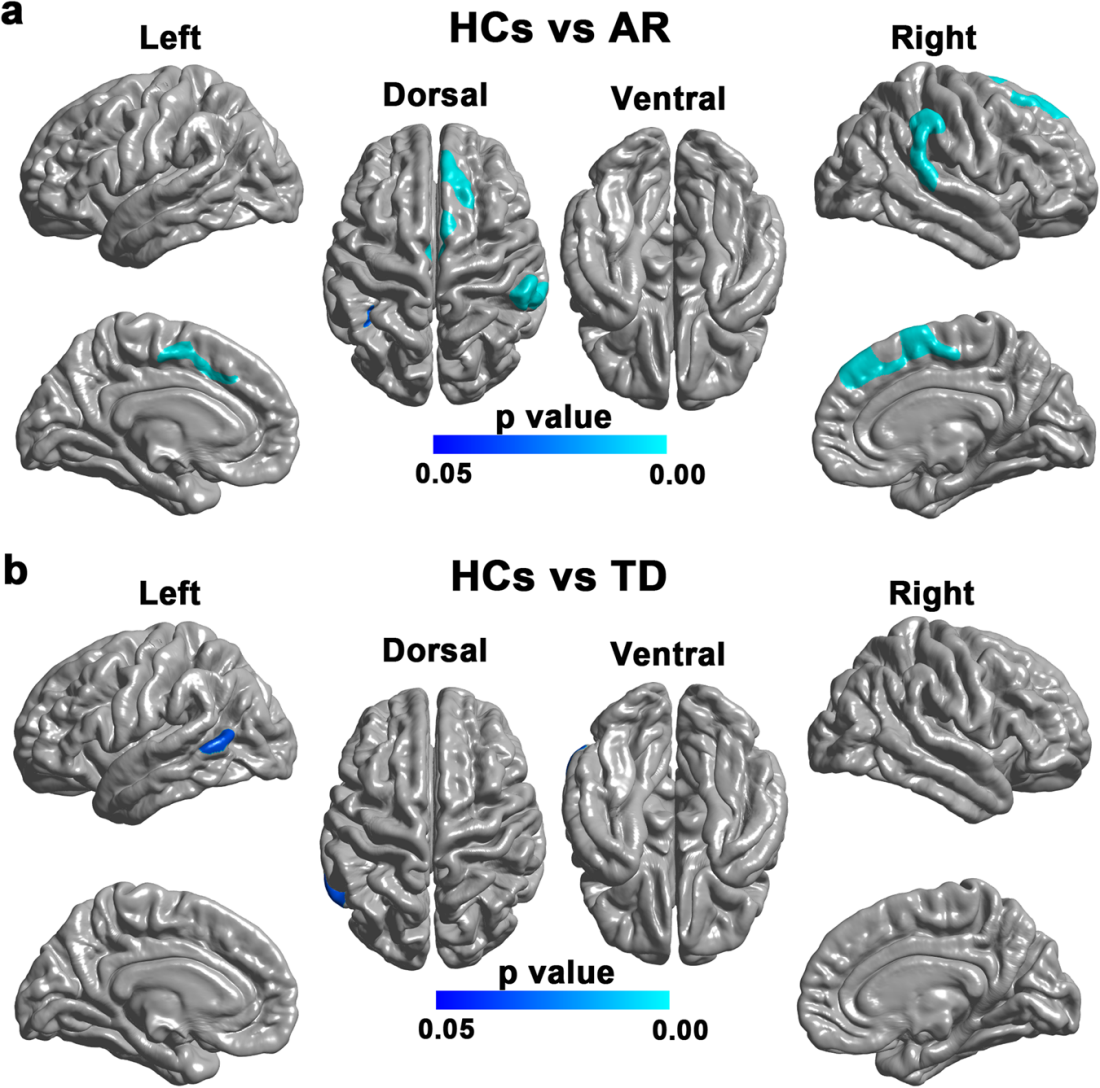
Brain regions with reduced surface area in the AR and TD subtypes compared with HCs. **a** Brain regions showing significantly reduced surface area in patients with the AR subtype compared with HCs. The result was corrected for multiple comparisons using RFT. The color bar indicates the RFT-corrected *p*-value. **b** Brain regions showing significantly reduced surface area in patients with the TD subtype compared with HCs. The result was corrected for multiple comparisons using RFT. The color bar indicates the RFT-corrected *p*-value.

Compared with HCs, patients with the AR subtype showed significantly decreased surface area in widespread cortical regions (Fig. 1a), including bilateral superior frontal gyrus, left paracentral lobule, left superior parietal lobule, right supramarginal and right superior temporal gyrus. Compare with HCs, patients with the TD subtype showed decreased surface area in the left posterior middle temporal gyrus (Fig. 1b). No significant difference was found in the surface area between the two PD subtypes.

### Sulc changes in AR and TD subtypes

**Fig. 2:**
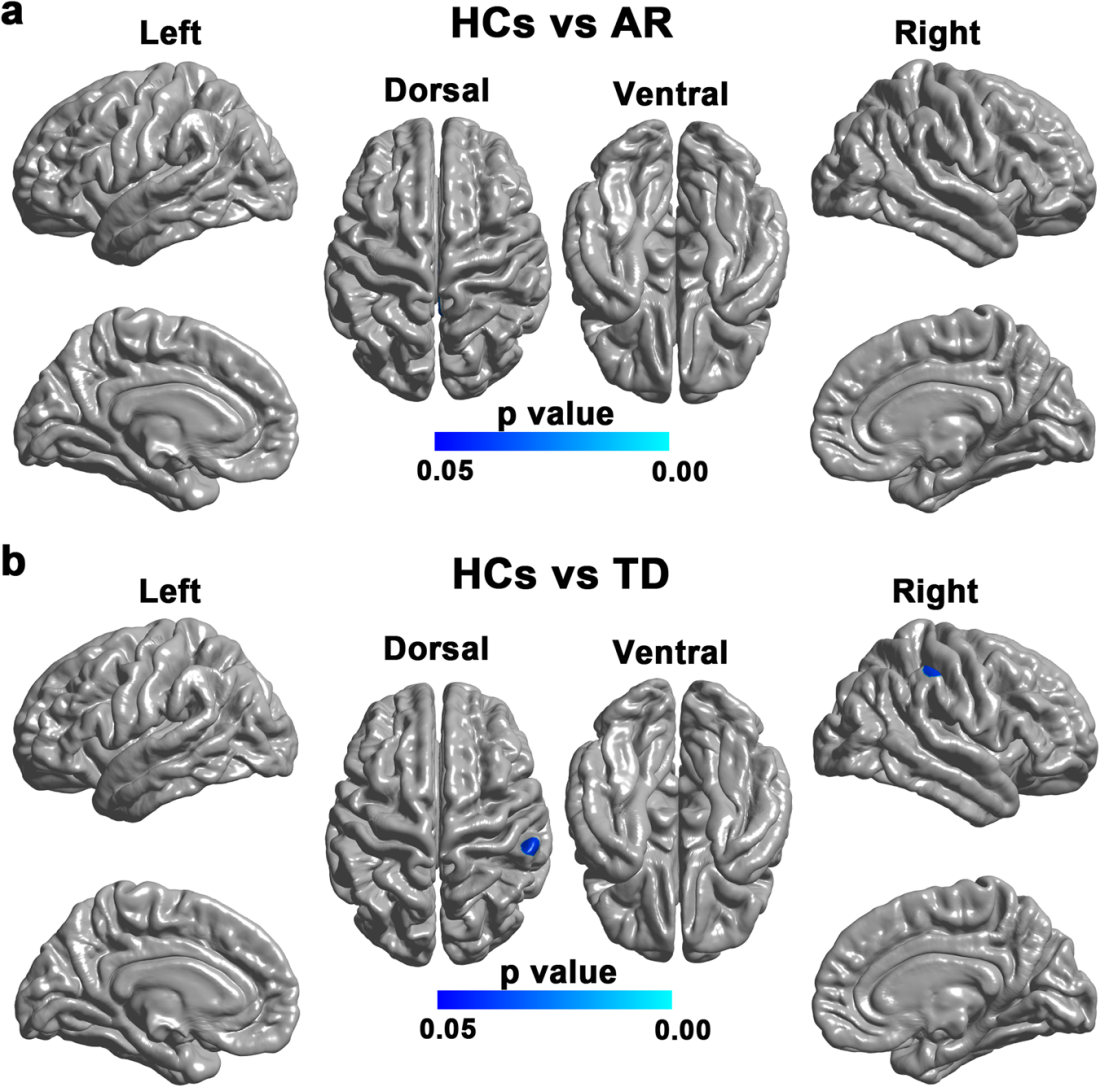
Brain regions with Sulc changes in the AR and TD subtypes compared with HCs. **a** Brain regions showing significant Sulc change in patients with the AR subtype compared with HCs. The result was corrected for multiple comparisons using RFT. The color bar indicates the RFT-corrected *p*-value. **b** Brain regions showing significant Sulc change in patients with the TD subtype compared with HCs. The result was corrected for multiple comparisons using RFT. The color bar indicates the RFT-corrected *p*-value.

Compared with HCs, we found no significant Sulc change in patients with the AR subtype (Fig. 2a) but revealed significantly higher Sulc in the right supramarginal gyrus in patients with the TD subtype (Fig. 2b). No significant difference was found in the Sulc between the two PD subtypes.

### Subcortical volumetric changes in AR and TD subtypes

Analyses of covariance for subcortical structures among the three groups revealed significant volumetric differences in bilateral hippocampi but not in other subcortical structures (false discovery rate-corrected *p* < 0.05) (Table 1). Specifically, the hippocampal volumes of the AR and TD subtypes were significantly lower than those of HCs, whereas there were no significant differences between the two subtypes.

**Table 1:** Volume of subcortical structures in PD patients and HCs.

Region	AR (mm^3^)	TD (mm^3^)	HCs (mm^3^)	*p*-value
Left hippocampus^a^	3781 ± 392	3651 ± 454	4017 ± 391	0.0005
Right hippocampus^a^	3994 ± 407	3890 ± 408	4198 ± 411	0.006
Left amygdala	1503 ± 205	1443 ± 253	1571 ± 195	0.085
Right amygdala	1718 ± 201	1654 ± 193	1742 ± 215	0.428
Left thalamus	6994 ± 649	6887 ± 805	7275 ± 932	0.154
Right thalamus	6767 ± 643	6738 ± 820	6946 ± 826	0.342
Left caudate	3225 ± 448	3109 ± 440	3286 ± 418	0.342
Right caudate	3305 ± 495	3236 ± 444	3295 ± 415	0.926
Left putamen	4556 ± 600	4590 ± 546	4657 ± 572	0.471
Right putamen	4555 ± 655	4653 ± 530	4716 ± 573	0.213
Left pallidum	2107 ± 232	2102 ± 234	1988 ± 226	0.013
Right pallidum	2103 ± 224	2079 ± 209	2004 ± 244	0.072
Left accumbens	347 ± 72	337 ± 85	362 ± 82	0.715
Right accumbens	430 ± 65	429 ± 78	454 ± 69	0.244

Data are mean ± standard deviation. The *p* values were obtained using one-way analysis of covariance while adjusting age and sex as covariates.

*AR* akinetic-rigid, *TD* tremor-dominant, *HCs* healthy controls.

^a^The cortical regions that survive the correction for multiple comparisons using false discovery rate approach.

### Relationships between morphological parameters and clinical data

**Fig. 3:**
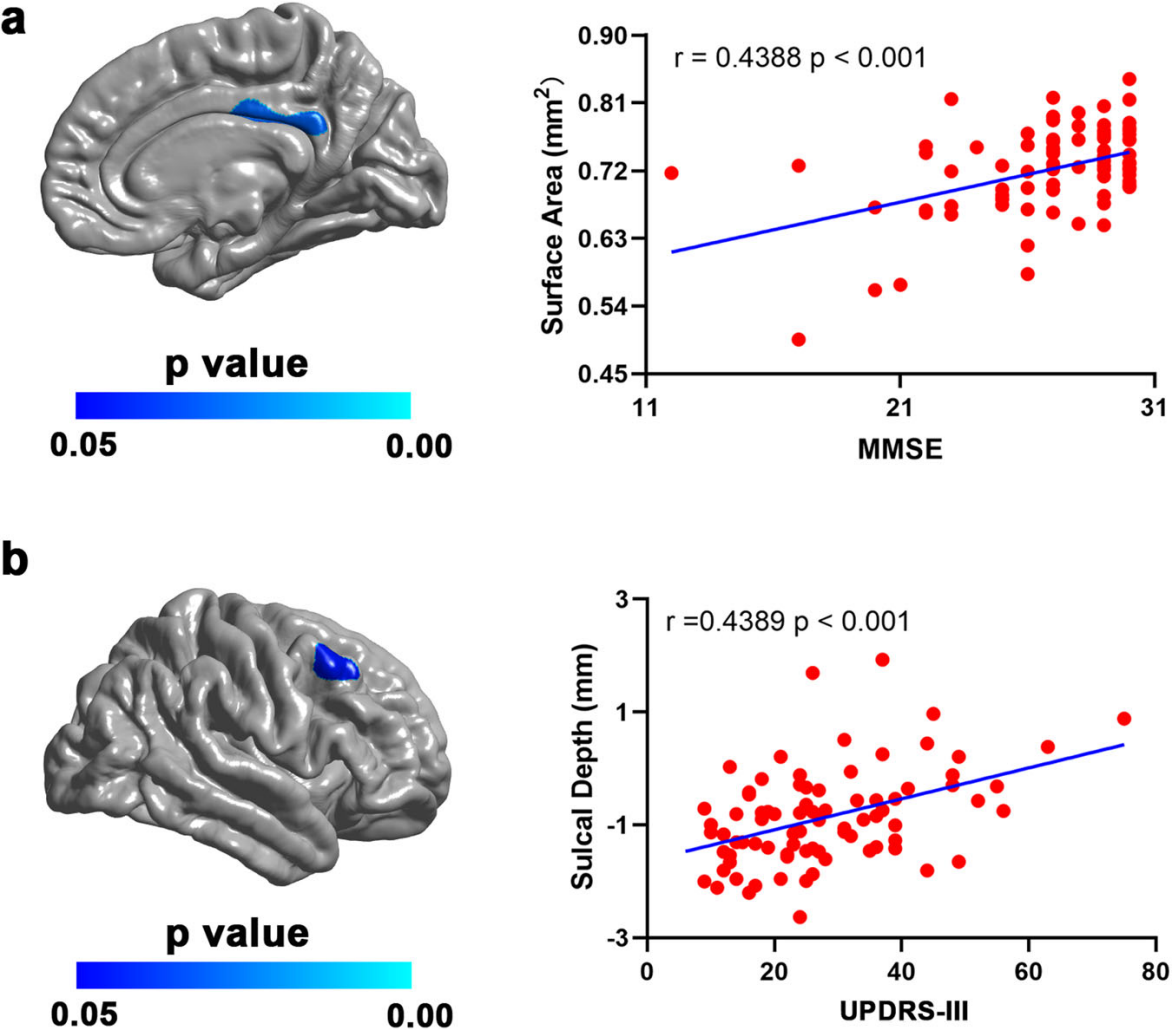
Relationships between cortical morphological parameters and clinical data in patients with PD. **a** The left panel shows the brain region where the surface area was positively correlated with the MMSE score in patients with PD. The result was corrected for multiple comparisons using RFT. The color bar indicates the RFT-corrected *p*-value. The right panel is a scatterplot of the positive correlation. **b** The left panel shows the brain region where the Sulc value was positively correlated with the UPDRS-III score in patients with PD. The result was corrected for multiple comparisons using RFT. The color bar indicates the RFT-corrected *p*-value. The right panel is a scatterplot of the positive correlation.

In patients with PD, the surface area of the posterior cingulate cortex was positively correlated with the Mini-Mental State Examination (MMSE) score (Fig. 3a), while the Sulc of the right middle frontal gyrus was positively correlated with unified PD Rating Scale (UPDRS-III) score (Fig. 3b). In addition, the volumes of bilateral hippocampi were positively correlated with the MMSE (Left: *r* = 0.3609, *p* = 0.0008; Right: *r* = 0.3271, *p* = 0.0024) and Montreal Cognitive Assessment (MoCA) (Left: *r* = 0.2819, *p* = 0.0078; Right: *r* = 0.2995, *p* = 0.0046) scores, and negatively correlated with the UPDRS-III (Left: *r* = −0.2179, *p* = 0.0391; Right: *r* = −0.2904, *p* = 0.0055) and H &Y (Left: *r* = −0.3186, *p* = 0.0023; Right: *r* = −0.2727, *p* = 0.0097) scores.

## Discussion

In this study, we examined the subtype-specific alterations in cortical and subcortical morphology in PD patients compared with HCs. We found more widespread surface area reductions in patients with the AR subtype than in patients with the TD subtype. In contrast, we found a focal Sulc increase in patients with the TD subtype but not the AR subtype. Subcortically, we found similar hippocampal atrophy in both AR and TD subtypes compared with HCs. In the patient group, we found significant positive correlations between the surface area of the posterior cingulate cortex and the MMSE score, and between the Sulc of the right middle frontal gyrus and the UPDRS-III score. In addition, the hippocampal volumes were found to correlate with the MMSE, MoCA, UPDRS-III, and H &Y scores. Taken together, these findings may contribute to a better understanding of the neural substrates underlying the distinct symptom profiles in the two PD subtypes.

We found more widespread surface area reductions in patients with the AR subtype relative to patients with the TD subtype. The pattern is, to some extent, in line with our previous surface-based analysis, which showed widespread reductions of local gyrification index in patients with the AR subtype but not in the TD subtype^9^. This finding is expectable as the TD subtype is considered a more benign subtype, often associated with milder clinical symptoms and a slower rate of progression^11^. Our finding of significant surface area reductions is also consistent with a recent multicenter neuroimaging study showing widespread surface area reductions in a large sample of patients with PD^12^. However, there were also studies reporting increased or unchanged surface area in patients with PD^13–15^. The exact mechanism for the inconsistency is unclear and may relate to the sample heterogeneity and methodological differences among studies.

Compared with HCs, patients with the AR subtype showed significantly decreased cortical surface area in the left superior parietal gyrus, the left paracentral lobule, and bilateral superior frontal gyrus in the AR subtype. These results are consistent with a previous study showing significant AR-related gray matter volume reduction and functional connectivity alterations in similar regions in PD patients^16^. The superior parietal gyrus has been involved in the integration of sensory and motor-planning processes as its anterior and posterior subregions are respectively connected with regions critically involved in sensory processing and executive functions^17,18^. The paracentral lobule and the mesial surface of the frontal lobe belong to the supplementary motor complex and play crucial roles in learning, sequencing, and executive control of movement^19,20^. As such, the observed surface area reductions in these areas may have contributed to the motor symptoms in the AR subtype. Compared with HCs, patients with the AR subtype also showed decreased surface area in the right supramarginal gyrus and right superior temporal gyrus, which are in agreement with previous reports of decreased local gyrification index^9^, functional nodal centralities^21^, and functional connectivity^22^ in these two regions in patients with PD. The supramarginal gyrus and neighboring superior temporal gyrus are located in the junction between the temporal, occipital and parietal lobules and have been shown to play critical roles in several cognitive processes such as social cognitive, spatial attention, language processing, and working memory^23–26^. The decreased surface area in these two regions may relate to the impaired cognitive functions in patients with the AR subtype. In addition, the present study also showed surface area reduction in the left posterior middle temporal gyrus in patients with the TD subtype compared with HCs. This result is in line with a previous study reporting an association between the volume atrophy in the middle temporal gyrus and the severity of tremor in patients with PD^27^. Given that the posterior middle temporal gyrus has been implicated in the planning and execution of actions, as well as the processing of semantic information^28,29^, the surface area reductions of this region in the TD subtype may play a role in the motor or cognitive deficits of this subtype. Taken together, the differential surface area reductions observed in the AR and TD subtypes may underlie the distinct symptom profiles in patients with the two motor subtypes.

Compared with HCs, the present study also found focal Sulc increase in the TD subtype but not in the AR subtype. Since the Sulc differences were observed in the crown of the supramarginal gyrus (having negative Sulc values), an increased Sulc value is equivalent to a lower absolute Sulc value and indicates a relatively lower crown of the supramarginal gyrus (and consequently a lower sulcal depth in nearby sulcal regions)^10^. This result is partially consistent with a previous study reporting significantly decreased sulcal depth in patients with PD^30^. The neural mechanisms underlying the increased Sulc values in the supramarginal gyrus however remain unknown and may relate to overlying gray matter thickness and white matter abnormalities^31,32^. Considering that the supramarginal gyrus has been shown to play important roles in various cognitive functions^24,26^, the observed Sulc increase in the right supramarginal gyrus in the TD subtype may be implicated in the cognitive dysfunctions of this subtype.

Compared with HCs, we found significant volumetric reductions in bilateral hippocampi in both AR and TD subtypes. This finding is consistent with previous reports of hippocampal atrophy in both motor subtypes^33,34^. Given that the hippocampus plays important roles in memory, navigation, and cognition^35–37^, the observed hippocampal atrophy may be associated with memory and cognition-related functional deficits in PD. This speculation is supported by the observed positive correlations of hippocampal volume with MMSE and MoCA scores in patients with PD in the present study as well as with intelligence quotient, memory^38,39^, and cognitive flexibility^34^ in healthy subjects and patients with various disorders in previous studies. In addition, we also revealed significant negative correlations between hippocampal volume and UPDRS-III and Hoehn and Yahr disability scale (H&Y) scores in patients with PD. The neural mechanism underlying such correlations is unclear since these measures assessed the motor impairments in PD. Considering the relationship between hippocampal volume and cognitive parameters (MMSE and MoCA scores), the negative correlation between hippocampal volume and the motor scores may suggest more severe cognitive impairments in patients with more severe motor disabilities^40,41^. Altogether, our findings may indicate that the observed hippocampal atrophy is likely due to worsening overall neurodegeneration, accounting primarily for the cognitive impairments rather than being a key driver of the motor deficits in PD.

In patients with PD, the surface area of the posterior cingulate cortex was found to be positively correlated with the MMSE score. This result was partially consistent with previous studies, showing significant correlations of the fractional anisotropy in the posterior cingulate cortex with various cognitive parameters including the Mattis Dementia Rating Scale, the revised version of Hasegawa’s Dementia Scale, and MMSE in PD^42^. The posterior cingulate cortex is a key node of the default mode network and has a central role in supporting internally-directed cognition^43^. The positive correlation between posterior cingulate surface area and the MMSE score may imply that the surface area of this region could be an indicator of cognitive capacity in patients with PD. Meanwhile, we also found a positive correlation between the Sulc of a cluster in the right middle frontal gyrus (i.e., the premotor cortex) and UPDRS-III score in patients with PD. Since this cluster is located in the crown of the middle frontal gyrus and has negative Sulc values, this finding indicates more severe motor deficits for patients with a lower gyrus of this region. The premotor cortex is anatomically connected with the prefrontal cortex, posterior parietal areas, primary motor cortex, and the spinal cord, and has been critically involved in the preparation, execution, and awareness of movement^44–46^. Hence, our finding of a positive correlation between the Sulc value of the premotor cortex and the UPDRS-III score suggests that the Sulc value of the middle frontal gyrus may serve as a marker for motor impairments in PD.

This study has several limitations that should be mentioned. First, the unbalanced sample sizes of the three groups might have confounded the conclusion drawn from the results, however depicting a naturalistic picture of real-life distribution. Future studies with a more balanced study design are needed to replicate current findings. Second, given that most of the PD patients were treated with dopaminergic medications, we could not rule out their effects on the brain morphologic alterations. Finally, the results of the cross-sectional study should be taken with caution considering the instability of motor subtype classifications over time^47,48^. Future longitudinal studies are warranted to characterize the dynamic profiles of the morphological alterations in the two motor subtypes.

In conclusion, this cross-sectional study found distinct patterns of alterations in the cortical surface area and sulcal depth yet a similar pattern of hippocampal atrophy in patients with the AR and TD subtypes compared with HCs. These findings may contribute to a better understanding of the neural substrates underlying the distinct symptom profiles in the two PD subtypes.

## Methods

### Participants

The present study included 90 PD patients and 56 HCs. These participants also took part in a previous study of our group^9^. Briefly, all the PD patients were diagnosed according to the UK Brain Bank criteria^49^ and completed the UPDRS, the Hoehn and Yahr disability scale, the Mini-Mental State Examination, and the Montreal Cognitive Assessment test in the OFF state. All participants underwent extensive neurologic, neuropsychological, and clinical imaging examinations. Using the formula published by Kang et al., the PD patients were categorized into AR and TD subtypes according to their UPDRS-III subitems^2^. Detailed demographics and clinical data are presented in Table 2. This study was approved by the Medical Ethical Committee of the Third Military Medical University. Written informed consent was obtained from all the participants.

**Table 2:** Demographic and clinical data of the PD patients and HCs.

	AR (*N* = 64)	TD (*N* = 26)	HCs (*N* = 56)	*p*-value
Age (years)	63 ± 10.3	60 ± 11.7	58.11 ± 8.0	0.071
Sex (male/female)	40/24	8/18	19/37	0.002
Duration of disease (years)	5.8 ± 4.2	7.6 ± 6.2	NA	0.170
Education (years)	8.9 ± 4.6	7.02 ± 3.7	NA	0.202
MMSE score	26.7 ± 3.7	27.2 ± 3.0	NA	0.297
MoCA score	20.8 ± 5.4	21.0 ± 6.5	NA	0.209
UPDRS-III score	29.1 ± 14.1	25 ± 11.2	NA	0.139
Hoehn and Yahr score	2.4 ± 0.7	2.15 ± 0.8	NA	0.072

Data represents means ± SD. The *p*-value for age was obtained by a one-way ANOVA test. The *p*-value for sex was obtained by chi-square test. The *p*-values for other variables were obtained by two-sample t-test.

*AR* akinetic-rigidity, *TD* tremor-dominant, *HCs* healthy controls, *MMSE* Mini-Mental State Examination, *MoCA* Montreal Cognitive Assessment, *UPDRS* unified Parkinson disease rating scale, *NA* not applicable.

### MRI data acquisition

All the T1-weighted MRI data were acquired on a Siemens 3.0 Tesla Tim Trio scanner with an eight-channel head coil using a three-dimensional magnetization-prepared rapid gradient-echo imaging sequence. The detailed scanning parameters are as follows: repetition time = 1900 ms; echo time = 2.52 ms; inversion time = 900 ms; flip angle = 9°; matrix = 256 × 256; thickness = 1.0 mm; voxel size = 1 × 1 × 1 mm^3^, 176 contiguous sagittal slices.

### Cortical surface area and Sulc maps

The T1-weighted brain MRI data of each participant was processed using FreeSurfer (https://surfer.nmr.mgh.harvard.edu/) to obtain the cortical surface area and Sulc maps. Specifically, the individual T1-weighted images were segmented to estimate the voxel-based gray/white matter boundary, which was triangulated to obtain a triangle-based gray/white matter boundary surface. This triangle-based gray/white matter surface was then topologically corrected to generate a refined gray/white matter surface. The resulting gray/white matter surface was further deformed outward with a deformable surface algorithm to generate the pial surface. Subsequently, the cortical surface area map of the pial surface was calculated by assigning one-third of the area of each triangle to each of its vertices. The Sulc map (lh/rh.sulc file of Freesurfer output) was calculated as a signed displacement from a vertex on the cortical surface to a hypothetical mid-surface that exists between the gyri and sulci. The Sulc map thus reflects how “deep” the sulci (with positive Sulc values) are and how “high” the gyri (with negative Sulc values) are. Prior to statistical analysis, the individual cortical surface area and Sulc maps were resampled onto the built-in “fsaverage” template of Freesurfer and further smoothed using a Gaussian kernel with a full-width-at-half-maximum of 20 mm.

Using the automated procedure for volumetric measurements of brain structures in FreeSurfer, we extracted the volumes of subcortical structures, including the thalamus, hippocampus, amygdala, putamen, globus pallidus, caudate, and accumbens.

### Statistical analyses

Vertex-wise contrasts of the cortical surface area and Sulc maps between the two patient groups and HCs were carried out using the SurfStat package (http://www.math.mcgill.ca/keith/surfstat/) in MATLAB. Specifically, for each vertex on the pial surface, we fitted a generalized linear model (GLM) with group, age, and sex as covariates. A vertex-wise *p* < 0.001 was used to define potential clusters of difference. Using random field theory (RFT), the resulting clusters were then corrected at the cluster level for multiple comparisons. The significance level for clusters was set at RFT-corrected *p* < 0.05.

Using the pooled data of the two patient groups, vertex-wise correlational analyses were carried out to examine the relationships between cortical maps and clinical data. In brief, a vertex-wise GLM was fitted with the variable of interest as a covariate. A vertex-wise *p* < 0.001 was used to define potential clusters, which were further corrected for multiple comparisons using RFT. The significance level for clusters was set at RFT-corrected *p* < 0.05.

Group differences in the volumes of each subcortical structure were tested using a one-way analysis of covariance after adjusting age and sex as covariates. The results were then corrected for multiple comparisons using the false discovery rate approach.

## Data Availability

The data used for this work are available from the corresponding authors upon reasonable requests such as reproducibility of research or external validation. Restrictions may be applied to sensitive data for privacy preservation.

## Code availability

The code used for this work is available from the corresponding authors upon reasonable requests such as reproducibility of research or external validation.
